# Genetic signals of high-altitude adaptation in amphibians: a comparative transcriptome analysis

**DOI:** 10.1186/s12863-016-0440-z

**Published:** 2016-10-03

**Authors:** Weizhao Yang, Yin Qi, Jinzhong Fu

**Affiliations:** 1 Chengdu Institute of Biology, Chinese Academy of Sciences, Chengdu, 610041 China; 2 Department of Integrative Biology, University of Guelph, Guelph, N1G 2 W1 ON Canada; 3 Present address: Department of Biology, Lund University, 223 62 Lund, Sweden

**Keywords:** Transcriptome, High altitude, Comparative analysis, Positive selection, F_ST_ outlier analysis, Nutrient metabolism, Amphibians, Asiatic toads

## Abstract

**Background:**

High-altitude adaptation provides an excellent system for studying how organisms cope with multiple environmental stressors and interacting genetic modifications. To explore the genetic basis of high-altitude adaptation in poikilothermic animals, we acquired transcriptome sequences from a high-altitude population and a low-altitude population of the Asiatic toad (*Bufo gargarizans*). Transcriptome data from another high-altitude amphibian, *Rana kukunoris* and its low-altitude relative *R. chensiensis*, which are from a previous study, were also incorporated into our comparative analysis.

**Results:**

More than 40,000 transcripts were obtained from each transcriptome, and 5107 one-to-one orthologs were identified among the four taxa for comparative analysis. A total of 29 (*Bufo*) and 33 (*Rana*) putative positively selected genes were identified for the two high-altitude species, which were mainly concentrated in nutrient metabolism related functions. Using SNP-tagging and F_ST_ outlier analysis, we further tested 89 other nutrient metabolism related genes for signatures of natural selection, and found that two genes, CAPN2 and ITPR1, were likely under balancing selection. We did not detect any positively selected genes associated with *response to hypoxia*.

**Conclusions:**

Amphibians clearly employ different genetic mechanisms for high-altitude adaptation compared to endotherms. Modifications of genes associated with nutrient metabolism feature prominently while genes related to hypoxia tolerance appear to be insignificant. Poikilotherms represent the majority of animal diversity, and we hope that our results will provide useful directions for future studies of amphibians as well as other poikilotherms.

**Electronic supplementary material:**

The online version of this article (doi:10.1186/s12863-016-0440-z) contains supplementary material, which is available to authorized users.

## Background

Understanding the genetic basis of adaptation is a major objective of modern evolutionary biology [1, 2], and organisms living in high-altitude environments provide some of the best study systems. Altitudinal gradients involve large ecological transitions over relatively short linear distances, and variations across such gradients provide strong evidence for selection driven local adaptation [3]. In addition, organisms at high-altitudes experience a multitude of stresses, such as low levels of oxygen, low temperature, high levels of UV radiation, and strong seasonality. Consequently, organisms require simultaneous adaptive responses to these challenges, which likely involve interactions and trade-offs between genes in their genetic networks [4]. This intertwined genetic basis of high-altitude adaptation offers excellent opportunities to explore the processes of adaptive evolution [4, 5].

Physiological adaptation or acclimatization to high-altitude environments has long been documented, and in some cases, its molecular genetic basis is also well understood. This is particularly true for endothermic vertebrates. In a low ambient environmental temperature, endotherms need to sustain metabolic heat production despite the reduced availability of oxygen. Subsequently, improved oxygen acquisition, transportation, and utilization are essential at high altitudes [5, 6]. At the molecular level, modifications of hemoglobin and the increased Hb oxygen affinity are arguably the best-studied adaptation to high altitudes [7, 8]. Recent genome-scan studies also revealed that genetic modifications associated with the hypoxia-inducible factor (HIF) pathway likely play a key role in Tibetan mammals such as the village dog, Tibetan human, Tibetan mastiffs, and yak [9–12]. Studies on Tibetan birds (e.g. the bar-headed goose and ground tit) also detected positive selection on genes involved in oxygen consumption [13, 14]. Other genetic pathways, such as oxidative phosphorylation (OXPHOS) that oxidizes nutrients and releases energy, are also well characterized among some high-altitude mammals and birds [13].

Poikilotherms are expected to have evolved different adaptive mechanisms at high altitudes compared to endotherms because of several fundamental physiological differences. Poikilotherms have much lower and variable body temperatures than homeothermic endotherms, and they do not use endogenous processes to maintain them. To survive long-term hypoxia, poikilothermic vertebrates are known to decrease metabolic demand and energy production, and hypothermia is often necessary in the process [15, 16]. In general, responses to high altitude conditions among poikilothermic vertebrates are much more variable and mechanisms are less understood [15]. For example, a survey of 27 South American lizards at various altitudes (0–4600 m) showed no correlation between their altitudinal range and key haematological parameters [17]. In contrast, high-altitude Andean frogs (genus *Telmatobius*, 3000–4200 m) have extremely high blood oxygen affinities and the smallest erythrocyte volume known in amphibians [18, 19]. Recent genome-scan studies on high-altitude poikilotherms also revealed a broad genetic response. Yang et al. [20, 21] examined a high-altitude ranid frog and a Tibetan agamid lizard. Several genes related to oxygen transport and the HIF pathway as well as response to UV damage, and a large number of genes associated with metabolic processes and gene expression regulation were identified as being under positive selection. Poikilotherms represent the majority of animal diversity, and more studies on them are needed to generate hypotheses that are applicable to a wide range of organisms.

The recent development of genomic technology makes genome-wide scans for non-model organisms readily feasible. Genome-scan, also known as the ‘reverse ecology’ approach, does not require *a priori* knowledge of adaptive phenotypes, and has potential to discover novel genetic mechanisms in adaptation studies compared to the traditional ‘candidate gene’ approach [22]. High-altitude adaptation requires coordinated changes in the regulation and structure of many genes, and genome-scan will likely achieve a more holistic understanding of high-altitude adaptation at the molecular level [4]. In the last few years, we have gained tremendous advances on the genetic mechanisms of high-altitude adaptation through this approach, especially for endothermic vertebrates [9, 10, 23]. Nevertheless, limits of the approach have also been recognized. Several processes, such as a small rate of sequencing error, demographic history, patterns of isolation by distance, and cryptic relatedness, can lead to false positives [24, 25]. Furthermore, designing experiments to assess the functional importance of true positives can be challenging, particularly for non-model organisms. Despite these limitations, the genome-scan approach has been applied to a wide range of species, and produced some of the most insightful clues that have later been verified by experiments (e.g. EGLN1 in human [26]).

Many amphibians have a large altitudinal distribution range and phenotypic differences along altitudinal gradients are well documented [27, 28]. At high altitudes, adult anurans tend to have a lower metabolic rate, lower developmental growth rate, larger body size, greater longevity than their low altitude relatives (although tadpoles often have different patterns); they also reach reproductive maturity at an older age, and produce fewer offspring per season [27–30]. Most of these variations have been attributed to low ambient temperature and shortening of annual active period [28]. The Asiatic toad (*Bufo gargarizans*) is one of the few amphibians living on the Tibetan Plateau. It has been a true Plateau dweller for approximately 2.5 Ma [31], and populations from high altitudes have shown significant differences from low-altitude populations. For example, Liao and Lu [29] found that adult toad populations from 2100 m had a slower growth rate and a delayed sexual maturity, but higher longevity and larger body size, compared to populations from 760 m. The species occupies an extremely large altitudinal gradient from 0 to 4300 m, which provides an excellent opportunity to compare individuals or populations from various altitudes.

In this study, we explored the genetic signals of high-altitude adaptation in the Asiatic toad (*Bufo gargarizans*) using a transcriptome-scan approach. Our specific objective is to identify genes that have likely experienced positive selection in high-altitude adult toad populations, with particular interests in genes or pathways that are closely related to regulating metabolism and oxygen transportation/consumption, which have been frequently identified in other animal species [9–14]. We acquired transcriptome sequences of individuals from both low- and high-altitude sites. With reference to other amphibian species, positive selection was tested. Furthermore, we examined 89 nutrient metabolism related genes along altitudinal gradients using SNP-tagging.

## Results

### Transcriptome sequence data

**Fig. 1:**
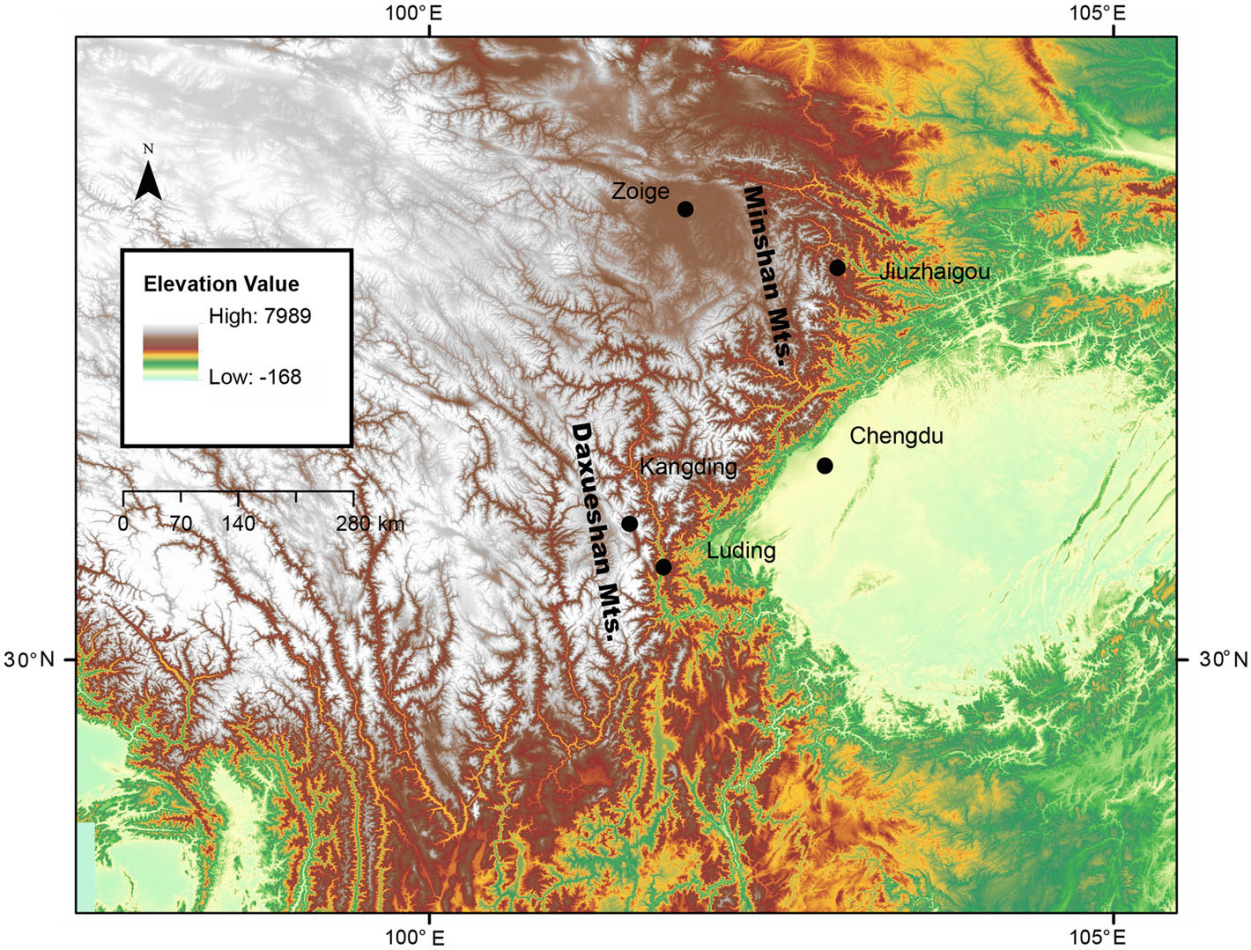
Map of western China with all sampling sites. For F_ST_ outlier analysis, 20 individuals were collected from each site. Three sites from the Minshan mountain range, Chengdu (559 m), Jiuzhaigou (1717 m), and Zoige (3464 m), form one altitudinal transect, and two sites from the Daxueshan mountain range, Luding (1465 m) and Kangding (3072 m), form the second transect. This map is created with ArcMap

We performed deep RNA sequencing (130 million reads, average coverage 250×) to minimize sequencing errors. Two high-quality transcriptome assemblies for the Asiatic toad were acquired, one from a low-altitude population (low-*Bufo*; Chengdu, 559 m) and the other from a high-altitude population (high-*Bufo*; Zoige, 3464 m; Fig. 1). A total of 40,959 transcripts were obtained for low-*Bufo*, with an N50 length of 1526 base pairs (bps) and a mean length of 1132 bps. Similarly, 49,194 transcripts were obtained for high-*Bufo* with an N50 length of 1606 bps and a mean length of 1103 bps.

Transcriptome sequences from another high-altitude anuran species, the plateau brown frog (*Rana kukunoris*; high-*Rana*), and its low-altitude relative, the Chinese brown frog (*Rana chensinensis;* low-*Rana*), were acquired from a previous study [20]. The two species are sister-species and diverged recently, and we included them in our analysis for comparison. The western clawed frog (*Xenopus tropicalis*), which is a lowland species and has the only well-annotated amphibian genome [32], was used as outgroup. A total of 5107 one-to-one orthologs were identified and used in downstream analyses.

### Tests for accelerated evolution

**Fig. 2:**
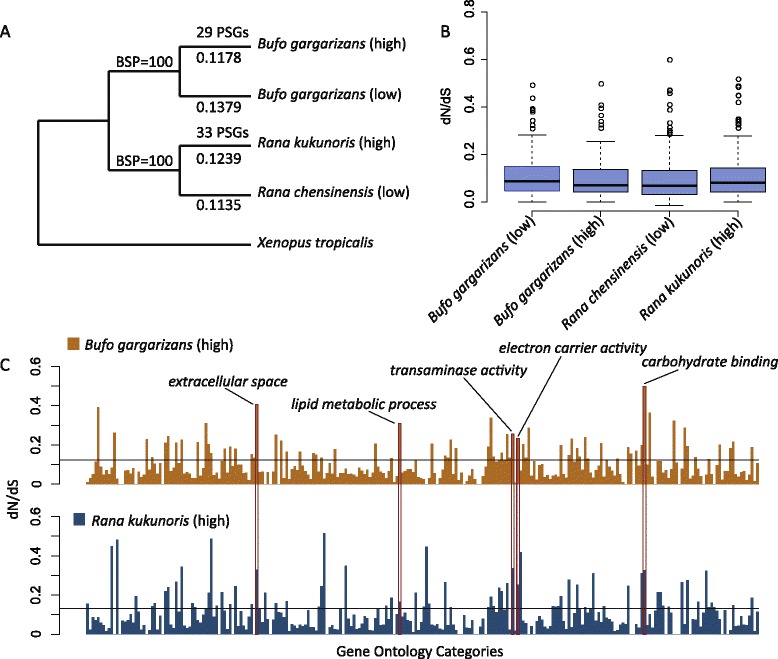
Summary results from comparative analysis of transcriptome sequence data. **a** Phylogenetic relationships of the study species. “High” indicates high-altitude lineages and “low” indicates low-altitude lineages. Numbers above the lines are numbers of putative positively selected genes (PSGs), and numbers below the lines are dN/dS ratios. Bootstrap proportions (BSP) from 1000 replications are also presented. **b** Distributions of dN/dS ratio estimated from 1000 bootstrap replications of the transcriptome-wide alignment for the four target branches. The high-altitude branches do not show significantly higher overall dN/dS ratios compared to their low-altitude relatives. **c** Average dN/dS ratios of gene clusters according to GO categories for the two high-altitude branches. Black lines represent the global average dN/dS ratios for each branch. High dN/dS categories shared by the two high-altitude lineages are marked by rectangles

A phylogenetic tree of the five taxa, low-*Bufo*, high-*Bufo*, low-*Rana*, high-*Rana*, and *X. tropicalis*, was constructed using the concatenated sequences of all orthologs and a maximum likelihood (ML) approach (Fig. 2a). The resulting topology was consistent with established amphibian phylogenies [33, 34].

We tested for accelerated evolution along the high-altitude branches. The dN/dS ratio was used to measure the evolutionary rate of coding genes, in view of their deep divergence [31, 35]. The ratios of the four ingroup branches varied from 0.1135 to 0.1379, and the two high-altitude branches revealed no accelerated evolution compared to their low-altitude relatives (binominal test, *P* > 0.05; Fig. 2b). Nevertheless, genes associated with certain functions demonstrated an accelerated evolution. Genes within five Gene Ontology (GO) categories had significantly higher dN/dS ratios than average in both high-altitude branches (FDR < 0.05), including *carbohydrate binding*, *electron carrier activity*, *extracellular space*, *lipid metabolic process*, and *transaminase activity* (Fig. 2c).

### Tests for positive selection

**Fig. 3:**
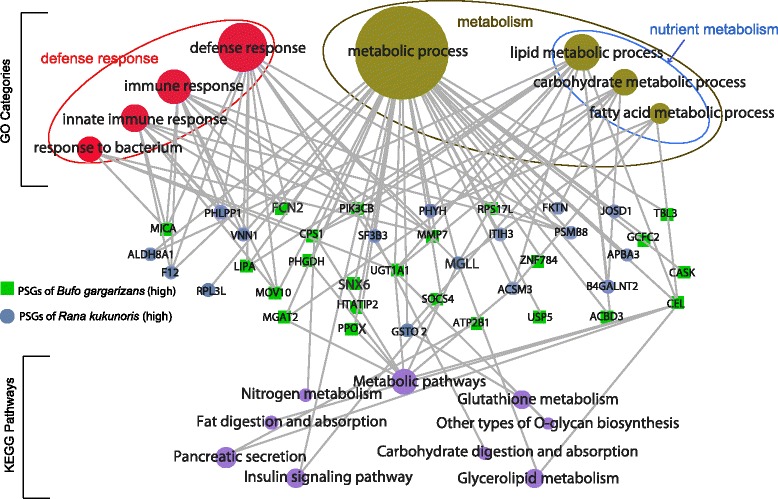
Genetic network of putative positively selected genes (PSGs) and their functions. Functions are defined using GO and KEGG annotations and network is constructed using the Rgraphviz package. Each solid circle or square represents a gene or a functional category. PSGs between the two species are very similar in functions and pathways. They were mostly concentrated in functions related to metabolism, especially nutrient metabolism, and defense response

We used the branch-site model to test for positive selection at specific loci along the high-altitude branches [36]. A total of 29 putative positively selected genes (PSGs) were identified along the high-*Bufo* branch (*P* < 0.05) (Fig. 2a), and 17 GO categories were over-represented (*P* < 0.05) (Additional file 1). A total of 33 putative PSGs were identified along the high-*Rana* branch, and 18 GO categories were over-represented (*P* < 0.05) (Additional file 1). The over-represented GO categories between the two high-altitude lineages were similar, and both included *defense response*, *immune response*, *lipid metabolic process*, and several others. Functional analysis using the Kyoto Encyclopedia of Genes and Genomes (KEGG) pathways revealed a similar pattern; several pathways related to metabolism were over-represented, such as *insulin signaling* and *fat digestion and absorption*. We constructed an integrated network for most PSGs and their GO and KEGG annotations for both high-altitude amphibians (Fig. 3). PSGs between high-*Bufo* and high-*Rana* revealed a strong similarity in GO categories and KEGG pathways, and they were mostly concentrated in functions related to immune response and metabolism, especially carbohydrate and lipid metabolic processes (Fig. 3). For instance, ACBD3, ACSM3, CEL, and LIPA are associated with lipid metabolic process, and PIK3CB and SOCS4 are part of the insulin-signaling pathway. Nevertheless, caution should be exercised. None of the above functional categories were significantly over-represented after correction for multiple tests (FDR > 0.05; Additional file 1).

### F_ST_ outlier analysis

**Fig. 4:**
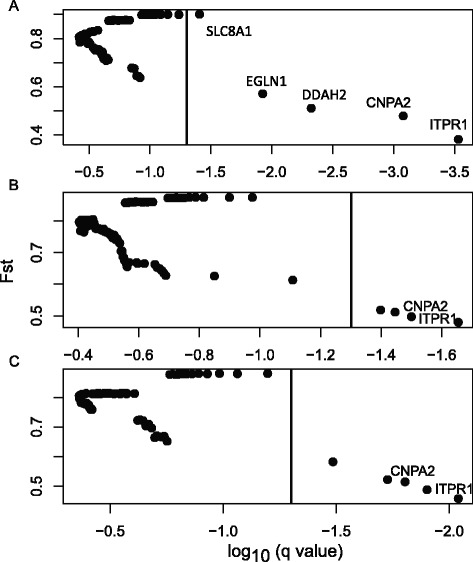
Results of the F_ST_ outlier analysis. BAYESCAN was used to generate the q values for each locus. Loci with q values of <0.05 are defined as outliers (on right side of the vertical line). A lower than expected FST value suggests balancing selection. Two loci, CAPN2 and ITPR1, are identified under balancing selection in all three tests. **a** Global test including all five populations. **b** Local test including the three populations along the Minshan Mountain transect. **c** Local test including the two populations of the Daxushan Mountain transect

We used SNP-tagging and an F_ST_ outlier method to further test natural selection on nutrient metabolism related genes in Asiatic toads. Population genetic methods are better at detecting recent positive selection, and therefore are complementary to the branch-site model [37]. We first selected 89 nutrient metabolism related genes based on GO and KEGG annotation, and then identified 101 tag SNPs for these genes based on our transcriptome sequence data (Additional file 2). A total of 100 individuals were genotyped, which were collected from five sites (20 individuals from each site) along two altitudinal gradient transects (Fig. 1). Three sites were from the Minshan mountain range with a maximum distance of 344 km and an altitudinal range of 559–3464 m, and the other two sites were from the Daxueshan mountain range with a distance of 63 km and an altitudinal range of 1465–3072 m. We found deep levels of divergence among populations, and the majority of F_ST_ values ranged between 0.6 and 0.9. These high F_ST_ values may have limited our ability to detect outliers that have higher than expected F_ST_. Using a Bayesian method implemented in BAYESCAN, we were able to identify five loci as F_ST_ outliers (q values <0.05), including CAPN2, DDAH2, EGLN1, ITPR1, and SLC8A1 (Fig. 4a; Additional file 3). SLC8A1 had the highest F_ST_ value of all loci, suggesting the gene may have recently experienced diversifying selection. The other four genes had lower than expected F_ST_ values, suggesting that they may have experienced balancing selection. We further tested F_ST_ outliers among sites along each transect. Loci that are detected in multiple independent inter-altitude comparisons are less likely results of false positive [38]. Two loci, CAPN2 and ITPR1, were consistently identified as outliers along both transects and had lower than expected F_ST_ values (Fig. 4). Both CAPN2 and ITPR1 are associated with calcium channel activity in energy metabolism [39, 40].

## Discussion

There are clear genetic signals of adaptation in high-altitude populations of Asiatic toads (*Bufo gargarizans*). Modifications of genes that are associated with nutrient metabolism (e.g. lipid metabolic process and insulin signaling) feature prominently and have likely played a major role in the adaptation process of adult toads.

We have identified nutrient metabolism related GO categories (e.g. lipid metabolic process, carbohydrate binding) that have accelerated evolutionary rates in both high-altitude amphibian species (Fig. 2). GO categories associated with lipid and carbohydrate metabolic processes are also particularly over-represented in PSGs (Fig. 3). Furthermore, our comparative study involves two species that represent two independent lineages, and the largely similar patterns between them further reinforce our conclusions. Amphibians at high altitudes generally have a short annual activity season with a cool and wild fluctuating temperature [27, 28]. For example, populations of *B. gargarizans* at Chengdu area (500 m) become active in early March, but populations at Zoige (3500 m) become active in early May [41]. The metabolism of amphibians largely depends on ambient temperature; with a cool and fluctuating temperature, modifications of their metabolism-related genes that allow their systems to function under these challenging conditions are probably beneficial. Additionally, shortage of food is a common challenge at high-altitude environments, and organisms must evolve adaptive strategies, such as pre-hibernation energy storage, to meet the challenge [42]. This challenge is likely more acute for amphibians because of their significantly shortened active period. Gene associated with nutrient metabolism were also identified as under positive selection in a Tibetan fish [43] and several Tibetan birds and mammals [10, 14, 44], although the pattern is much less pronounced in endotherms. Additionally, population level analysis identified two genes (CAPN2 and ITPR1) that are likely under balancing selection. Although both genes are functionally related to energy metabolism [39, 40], how balancing selection on them may contribute to high-altitude adaptation is unclear.

Several genes associated with immune functions and defense response are identified as PSGs (Fig. 3). Immune related genes are generally subjected to a wide range of selection pressures, in particular host-parasite interaction, and are commonly found under positive selection during processes of divergence [45–47]. Therefore, these PSGs may not be directly related to adaptation to high-altitude environments. Nevertheless, the immune functions of ectotherms are strongly influenced by ambient temperature and other environmental stressors [48]. Contribution of immunity related genes to high-altitude adaptation remains to be explored.

We did not detect any positively selected genes associated with *response to hypoxia*, and this represents a significant difference from endotherms. Hypoxia is a major environmental stressor at high altitudes and a large number of genes associated with hypoxia, particularly genes of the HIF pathway, experienced positive selection in several Tibetan endotherms, including the ground tit, Tibetan human population, and yak [10, 14, 23]. Instead, we detected a weak signal of balancing selection for EGLN1 (Fig. 4), which is a key component of the HIF pathway. It is a little surprising and difficult to explain that the gene is under balancing selection, not diversifying selection. Nevertheless, the signal appeared only in one test, not the other two (Fig. 4; Additional file 3), and therefore, the results could be a false positive [38]. There are several potential causes of the lack of PSGs associated with *response to hypoxia*. For the F_ST_ outlier analysis, we had very high F_ST_ values (0.6–0.9), which have likely limited our ability to detect outliers that have higher than expected F_ST_. Also, whether the HIF is an important organizer of hypoxia response in poikilothermic vertebrate remains unresolved [15]. It is an interesting question for future research.

Our results are consistent with existing phenotypic and physiological evidence. In laboratory experiments, poikilothermic vertebrates suppress their metabolism to survive in hypoxia and hypothermia, hence reducing their oxygen demands [15]. Rather than improve their oxygen uptake, high-altitude poikilotherms may decrease their metabolism while maintaining normal physiological activities. Amphibians are known for having the lowest resting metabolic rates and lowest energy requirements of any terrestrial vertebrates [28]. In addition, an array of amphibians exhibited a decreased growth rate along with the increase in altitude, including the Asiatic toad [29], *Bufo bufo* [49], and *Nanorana parkeri* [50]. Growth rate is often positively correlated with metabolic rate and nutrition supply [27, 51]. Reduced growth rates suggest low metabolic rates and low nutrition uptake. Modifications at gene sequence level that we detected are likely associated with these physiological changes. Nevertheless, functional validation is required to establish such associations.

There are several limitations of our study. First, we only examined the transcriptomes of adults. Most amphibians have a two-phase life cycle, an aquatic larval phase (tadpoles) and a terrestrial adult phase, and tadpoles and adults often developed different adaptive strategies to survive [28]. For example, some high-altitude tadpoles have faster development and growth rates than the low-altitude larvae, which is likely the result of a counter-gradient selection [49, 52, 53]. Tadpoles express many different genes compared to adults. To better understand the adaptation of amphibians, tadpole transcriptomes should be examined to complement the studies of adults. Second, plasticity, such as increases in hematocrit and Hb concentration as well as differential expression of genes related to aerobic metabolism, plays an important role in high-altitude adaptation [5]. In addition to modifications at sequence level, adaptive variations at gene expression level should also be explored. Last, genome-wide scanning generates interesting hypotheses; however, these hypotheses need to be corroborated with further biochemical and physiological studies. Without such corroboration, such hypotheses can serve only as suggestions. In order to make meaningful contributions to our understanding of the molecular mechanisms of high-altitude adaption, the candidate genes detected in our study need to be validated using functional analysis in future studies [54–56].

## Conclusions

Amphibians likely employ different genetic mechanisms for high-altitude adaptation compared to endotherms. Modifications of genes associated with nutrient metabolism feature prominently while genes related to hypoxia tolerance may not be so important. Poikilotherms represent the majority of animal diversity, and we hope that our results will provide useful directions for future studies of amphibians as well as other poikilotherms.

## Methods

### Sample collection

For transcriptome sequencing, samples of Asiatic toads were collected from a low-altitude site (Chengdu, China, 104.01°E, 30.91°N, 559 m) and a high-altitude site (Zoige, China, 102.48°E, 33.72°N, 3464 m; Fig. 1). Eight individuals (four males and four females) were collected from each site by hand, and six different tissues (brain, liver, heart, muscle, and testicle/ootheca) were collected from each individual. Tissue samples were stored in Sample Protector (*Takara*) immediately following euthanasia and dissection.

Samples for SNP genotyping were collected from five sites along two altitudinal gradient transects, and 20 individuals were captured from each site. Three sites, Chengdu (104.01°E, 30.91°N, 559 m), Jiuzhaigou (104.15°E, 33.08°N, 1717 m), and Zoige (102.48°E, 33.72°N, 3464 m) are located in the Minshan mountain range and form the first transect. Two sites, Luding (102.24°E, 29.80°N, 1465 m) and Kangding (101.87°E, 30.27°N, 3072 m), are located in the Daxueshan mountain range and form the second transect. A toe from each individual was collected and preserved in 95 % ethanol. A map with all sampling sites is presented in Fig. 1.

### Transcriptome sequencing and assembly

RNA was extracted separately from each tissue according to the TRIzol protocol (Invitrogen) and all RNA from the same site was pooled with approximately same quantity. A single cDNA library was constructed for each site and subsequently sequenced on an Illumina HiSeq 2000 platform. Paired-end sequencing was conducted with a read length of 100 base pairs (bps). Both cDNA library construction and Illumina sequencing were carried out by BGI (Shenzhen, China). The raw sequence reads were first cleaned by filtering adapter sequences, sequences with unknown base call (N) more than 5 %, low quality sequences (<Q20 [57]), as well as exact duplicates produced by sequencing from both directions. Reads likely derived from contaminants of *Escherichia coli* and human were also filtered out using Bowtie [58]. *De novo* assembly of clean reads was performed using a combination of five K-mer lengths and six coverage cut-off values using ABYSS [59]. A total of 30 raw assemblies were first constructed and a final assembly was created by integrating sequence overlaps and eliminating redundancies [20].

### Orthologous inference

Genomic data from three additional species, *Rana chensinensis*, *R. kukunoris*, and *Xenopus tropicalis*, were included in our analysis. Transcriptome data of the *Rana* species were obtained from NCBI Sequence Reads Archive (SRA060325), and coding sequences of *X. tropicalis* were extracted from its genome data in bioMart (Ensembl Genes 74). A best reciprocal hit (BRH) method [60] was used to identify one-to-one orthologs using tBlastx with the e-value threshold of 1e-10. The identified orthologous sequences were aligned using the “codon alignment” option in Prank [61], and the alignments were further trimmed using Gblocks [62] to remove unreliable regions with “codon” option (“-t = c”) and the default parameters. A saturation test was performed for each ortholog to remove sequences with saturation at synonymous sites. When synonymous substitutions are saturated, dN/dS ratio has a tendency of being over-estimated, which may cause false positives when identifying positively selected genes [63]. Sequences with unexpected stop codons and with alignment length less than 200 bps were discarded to reduce the chance of false positive prediction.

### Phylogenetic construction and test for accelerated evolution

A phylogenetic tree of *Bufo gargarizans* (high-altitude), *B. gargarizans* (low-altitude), *Rana chensinensis*, *R. kukunoris*, and *Xenopus tropicalis* was constructed using the concatenated sequences of all orthologs. A maximum likelihood (ML) analysis was carried out using RAxML [64] with GTR + R model and 1000 bootstrap replicates. Based on the resulting phylogeny, we examined the evolutionary rate for each branch using a branch model in the program CODEML (in the PAML4 package [36]). The ratio of the number of non-synonymous substitutions per non-synonymous site (dN) to the number of synonymous substitutions per synonymous site (dS) was used to measure the evolutionary rate. A distribution of the dN/dS ratio was generated for each branch by 1000 replicates of bootstrapping, and a binominal test was used to test significant rate differences between the high-altitude lineages and their low-altitude relatives.

### Test for positive selection with the branch-site model

Based on the well-established phylogenetic hypothesis for these five taxa, an optimized branch-site model implemented in CODEML [36] was used to identify positively selected genes (PSGs). The high-*Rana* and high-*Bufo* lineages were separately set as the foreground branch. A likelihood ratio test (LRT) was conducted to compare the model with positive selection to a null model with neutral evolution on the foreground branch for each ortholog. Putative PSGs were inferred only if their *P* values were less than 0.05.

### Test for selection with an F_ST_ outlier method

Only genes associated with nutrient metabolism were subjected to this set of analysis. Candidate genes were first identified according to GO and KEGG annotation. SNP sites were then identified by mapping the clean reads to the transcriptome assembly of high-*Bufo* using Bowtie [58] and SAMtools pipeline [65]. No insertion or deletion variants were considered, and a putative SNP site was inferred only if the allele coverage was greater than 20 for rare alleles.

Genomic DNA was extracted by the phenol/chloroform method from each toe tissue sample and all putative SNPs were genotyped by the MALDI-TOF Mass Spectrometry in Sangon Biotech (Shanghai, China). Within each population, SNP loci were tested for departure from Hardy-Weinberg equilibrium using ARLEQUIN 3.5 [66] with the Markov Chain (MC) length of 10^6^ and 100,000 dememorizations. All loci were also tested for linkage disequilibrium using GENEPOP 4.0 [67] with 10,000 dememorizations, 100 batches, and 5000 iterations.

A Bayesian method, implemented in BAYESCAN 2.1 [68], was used to identify F_ST_ outliers, which are characterized by higher or lower levels of population differentiation than strictly neutral loci. For each locus, BAYESCAN calculates a posterior probability for a model that includes selection. It also estimates a q value and an alpha value for each locus. FDR is used by the program to correct for multiple tests, and the q value is the FDR analogue of P value. An alpha significantly different from zero indicates departure from neutrality; a positive alpha suggests diversifying selection while a negative alpha suggests balancing selection. We used a q of <0.05 to define outliers and used F_ST_ and alpha values to determine types of selection. Three tests were conducted separately, a global test included all five populations, and two local tests included samples along each of the two transects. Local tests involved only sites within a short linear geographic distance, which would minimize potential impacts of isolation by distance.

## Additional files

Additional file 1: List of putative positively selected genes (PSGs) in high-altitude Asiatic toads (*Bufo gargarizons*) and the plateau brown frog (*Rana kukunoris*), as well as the Gene Ontology categories over-represented (*P* < 0.05) by these PSGs. (XLSX 17 kb) Additional file 2: List of the 89 nutrient metabolism associated genes and their tag SNPs. These candidate genes are identified according to GO and KEGG annotation and are subjected to F_ST_ outlier analysis. (XLSX 13 kb) Additional file 3: Results of F_ST_ outlier analysis and the testing parameters from BAYESCAN. The q value is the FDR analogue of *P* value. A positive alpha suggests diversifying selection while a negative alpha suggests balancing selection. (XLSX 11 kb)

## Abbreviations

ACBD3: Acyl-CoA binding domain containing 3
ACSM3: Acyl-CoA synthetase medium-chain family member 3
bp: Base pair
CAPN2: Calpain 2
CEL: Carboxyl ester lipase
DDAH2: Dimethylarginine dimethylaminohydrolase 2
dN: Number of non-synonymous substitutions per non-synonymous site
dS: Number of synonymous substitutions per synonymous site
EGLN1: Egl-9 family hypoxia-inducible factor 1
FDR: False discovery rate
GO: Gene ontology
HIF: Hypoxia-inducible factor
ITPR1: Nositol 1,4,5-trisphosphate receptor, type 1
KEGG: Kyoto Encyclopedia of Genes and Genomes
LIPA: Lipase A
LRT: Likelihood ratio test
ML: Maximum likelihood
OXPHOS: Oxidative phosphorylation
PIK3CB: Phosphatidylinositol-4,5-bisphosphate 3-kinase, catalytic subunit beta
PSG: Positively selected genes
SLC8A1: Solute carrier family 8, member 1
SNP: Single nucleotide polymorphism
SOCS4: Suppressor of cytokine signaling 4
